# Neutralization of interleukin-17 suppresses allergic rhinitis symptoms by downregulating Th2 and Th17 responses and upregulating the Treg response

**DOI:** 10.18632/oncotarget.15652

**Published:** 2017-03-01

**Authors:** Zhao Wei Gu, Yun Xiu Wang, Zhi Wei Cao

**Affiliations:** ^1^ Department of Otorhinolaryngology, China Medical University Affiliated Shengjing Hospital, Shenyang, Liaoning, China; ^2^ Department of Medical Insurance, China Medical University Affiliated Shengjing Hospital, Shenyang, Liaoning, China

**Keywords:** interleukin-17, neutralization, Th2, Th17, allergic rhinitis, Immunology and Microbiology Section, Immune response, Immunity

## Abstract

Allergic rhinitis (AR) has long been considered to predominantly involve the actions of Th2 cells, with relatively small contributions from Th1 cells. In recent years, the discovery of Th17 and regulatory T (Treg) cells has rendered the Th1/Th2 balance paradigm more complex and expanded our understanding of the pathogenesis of AR. IL-17, a key cytokine produced by Th17 cells, is known to induce allergen-specific Th2 cell activation, eosinophil and neutrophil accumulation, and serum IgE production in asthma; all of these features may play important roles in AR. To the best of our knowledge, only a few studies have assessed the feasibility of using IL-17 antagonists to treat AR. Thus, the principal objectives of the present study were, first, to determine the status of Th17 and Treg cells in the nasal mucosa of a mouse model of AR, and, second, to investigate the effects of IL-17 on such cells and the therapeutic efficacy of anti-IL-17 antibodies (Abs) in the context of AR. Anti-IL-17 Abs were given intranasally during the re-challenge of BALB/c mice with ovalbumin (OVA)-induced AR. We measured the numbers of nasal rubbing motions and sneezes, eosinophil and neutrophil levels, Th1, Th2, Th17, and Treg parameters in the nasal mucosa. Anti-IL-17 Abs markedly reduced the number of nasal rubbing motions and sneezes, decreased eosinophil and neutrophil infiltration, reduced Th2 and Th17 responses, and increased the Treg response. Anti-IL-17 Ab treatment protects against AR. These results will improve our understanding of AR pathogenesis and may lead to the development of novel therapeutic approaches for management of the condition.

## INTRODUCTION

Allergic rhinitis (AR) is an allergic inflammatory reaction of the nasal mucosa; nasal symptoms include sneezing, rhinorrhea, nasal blockage, and scratching of the nose [1]. The incidence of AR has gradually increased in recent years. Although AR is not life-threatening, it seriously impacts the quality of life and work efficiency of affected individuals.

AR has long been considered to involve predominantly Th2 cells; any contribution by Th1 cells was considered low [2]. In recent years, the discovery of Th17 and regulatory T (Treg) cells has added additional layers of complexity to the older Th1/Th2 balance paradigm, and it has expanded our understanding of the pathogenesis of AR [3].

Th17 cells were first identified as IL-17-producing cells in the year 2000 [4], and a relevant mouse transcription factor, RORγt, was discovered in 2006 [5]. Treg cells express the specific transcription factor Foxp3 and play a key role in preventing immune activation, downregulating factors triggering lesional inflammation [6].

On allergen sensitization, Th17 cells enhance not only neutrophilic airway inflammation but also Th2 cell-mediated eosinophilic airway inflammation in mouse models of asthma [7, 8]. Previous studies showed that Foxp3 mRNA expression was reduced in AR patients [9–11]. However, the precise contributions of Th17 and Treg cells to the nasal mucosal status of AR patients have not been thoroughly investigated and thus remain controversial.

IL-17 is a key cytokine produced by Th17 cells. Serum IL-17 levels are increased in AR patients and are associated with clinical symptoms, peripheral eosinophil counts, and the need for medication [12, 13].To the best of our knowledge, few studies have explored whether an IL-17 antagonist would be effective to treat AR [14]. As Th17 and/or Treg cells and IL-17 may play important roles in AR, we hypothesized that anti-IL-17-neutralizing antibodies (Abs) might be protective.

Thus, our principal objectives were, first, to determine the nasal mucosal status of Th17 and Treg cells in a mouse model of ovalbumin (OVA)-induce AR, and, second, to explore whether anti-IL-17 Abs affected the activities of such cells, to ameliorate AR.

## RESULTS

### IL-17 inhibition reduces nasal symptoms in an AR mouse model

**Figure 1:**
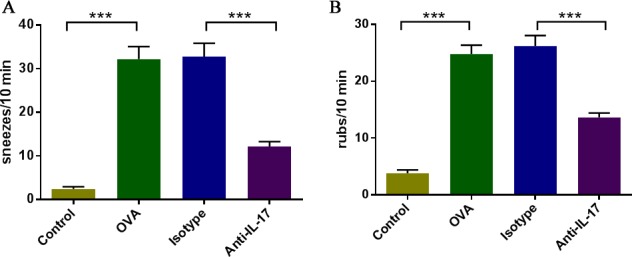
IL-17 inhibition ameliorated nasal symptoms **A**. Sneezing symptom scores. **B**. Rubbing symptom scores. *** *P* < 0.05.

We evaluated the effects of anti-IL-17 Abs on AR symptoms in mice. On day 25 of the experiment, during the 10-min period after the final intranasal OVA administration, the numbers of sneezes and nasal rubs were counted (Figure 1). The OVA-induced mice sneezed significantly more frequently than did the mice in control group (*P* < 0.05). Mice treated with anti-IL-17 Abs (anti-IL-17 group) sneezed significantly less frequently than did mice treated with isotype Abs (isotype group) (*P* < 0.05). Increased nasal rubs in OVA-induced mice were diminished by treatment with anti-IL-17 Abs, but not with isotype Abs. Thus, anti-IL-17 treatment suppressed AR symptoms.

### IL-17 inhibition reduces eosinophil numbers

**Figure 2:**
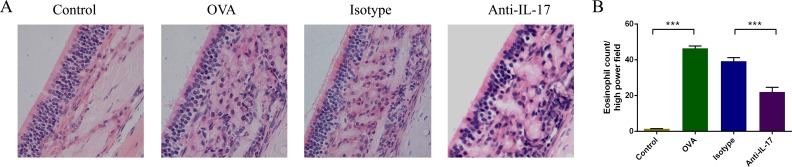
Anti-IL-17 Abs reduced eosinophil infiltration into the nasal mucosa Representative photomicrographs (original magnification ×200) of sections of mouse nasal mucosa stained with H&E to allow eosinophil visualization (red color) **A**. Significantly increased numbers of eosinophils **B**. were evident in the OVA group compared to the control group, and this increase was obviously alleviated by anti-IL-17 Ab treatment. *** *P* < 0.05.

To evaluate the effects of anti-IL-17 Abs on eosinophil numbers in OVA-induced AR mice, mice were sacrificed 2 h after the last OVA challenge on day 25 of the experiment and their nasal mucosae evaluated after H&E staining (red) of the cytoplasm of eosinophils (Figure 2A). Significantly more eosinophil infiltration was evident in OVA group than in control group (*P* < 0.05). Upon treatment with anti-IL-17 Abs, eosinophilic infiltration decreased significantly compared to treatment with isotype Abs (*P* < 0.05) (Figure 2B). These results indicate that anti-IL-17 Ab treatment inhibited eosinophil infiltration.

### IL-17 inhibition reduces neutrophil numbers

**Figure 3:**
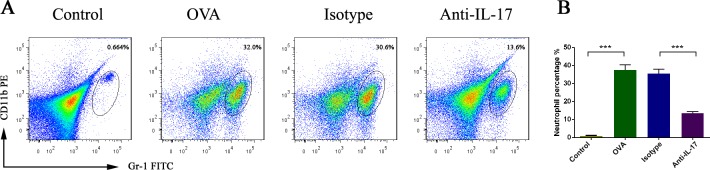
Anti-IL-17 Abs reduced neutrophil infiltration into the nasal mucosa All data are representative of the results of four independent experiments **A**. The statistics are shown in **B**. The numbers in the upper right quadrants are the proportions of neutrophils (B). *** *P* < 0.05.

Neutrophil accumulation was linked mechanistically to the effects of IL-17. We evaluated the effect of anti-IL-17 Abs on neutrophil by flow cytometry (Figure 3). The proportion of neutrophils increased in OVA-induced mice than the control mice (*P* < 0.05). After treatment with anti-IL-17 Abs, this proportion fell significantly (*P* < 0.05). These results indicate that anti-IL-17 Ab treatment inhibited neutrophils infiltration.

### IL-17 inhibition changes cytokine levels in the nasal mucosa

**Figure 4:**
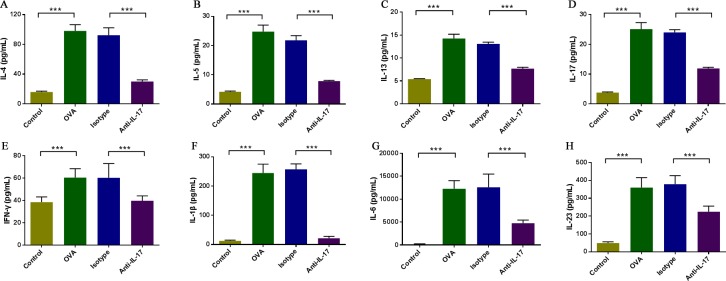
Anti-IL-17 Abs decreased the levels of cytokine levels in nasal mucosa The protein levels of IFN-γ, IL-1β, IL-4, IL-5, IL-6, IL-13, IL-17 and IL-23 were analyzed using a CBA. Compared to the control group, the nasal mucosal levels of all cytokines were significantly elevated in the OVA group, and the administration of anti-IL-17 Abs markedly reduced the levels of IL-4, IL-5, IL-13, IL-17, IFN-γ, IL-1β, IL-6, and IL-23 (A, B, C, D, E, F, G, and H, respectively). *** *P* < 0.05.

To evaluate the effects of anti-IL-17 Ab treatment on Th1, Th2 and Th17 cell-related cytokine levels in the nasal mucosa, the protein levels of IFN-γ, IL-4, IL-5, IL-13, and IL-17 were analyzed using a CBA (Figure 4A-4E). Compared to the control mice, the levels of IFN-γ, IL-4, IL-5, IL-13, and IL-17 in the nasal mucosa were significantly increased in OVA-induced mice (*P* < 0.05), and such up-regulation was markedly inhibited upon anti-IL-17 Ab treatment (*P* < 0.05). Thus, the blockade of IL-17 significantly suppressed the levels of IFN-γ, IL-4, IL-5, IL-13, and IL-17, indicating that the suppression of nasal mucosal inflammation by anti-IL-17 Abs is attributable to decreases in the levels of multiple inflammatory mediators.

Then, we evaluated the effects of anti-IL-17 Abs on the levels of IL-1β, IL-6 and IL-23 (Figure 4F-4H), which played important role in promotion of Th17 differentiation and development.

The levels of IL-1β, IL-6 and IL-23 were significantly increased in OVA-induced mice than the control mice (*P* < 0.05), and such up-regulation was markedly inhibited upon anti-IL-17 Ab treatment (*P* < 0.05). These results indicate that anti-IL-17 Ab treatment inhibited pro-inflammatory mediators for Th17 cells.

### Effect of an IL-17 blockade on the levels of mRNAs encoding Th1, Th2 and Th17 cell-related cytokines and Th1, Th2, Th17, and Treg cell-specific transcription factors in nasal mucosa

**Figure 5:**
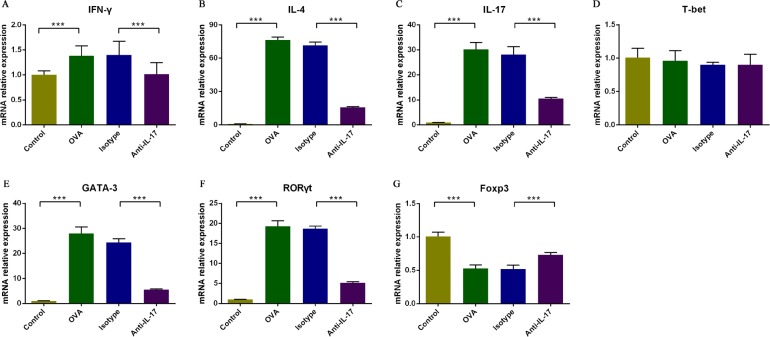
Analysis of the effects of an IL-17 blockade on the nasal mucosal expression of mRNAs encoding Th1, Th2 and Th17 cell-related cytokines and of Th1, Th2, Th17, and Treg cell-specific transcription factors using real-time PCR The relative mRNA expression levels of IFN-γ, IL-4, IL-17, T-bet, GATA-3, and RORγt **A**., **B**., **C**., **D**., **E**., and **F**, respectively) were significantly upregulated in the OVA group compared with the control group, and such upregulation was markedly inhibited by anti-IL-17 Abs. Compared to the control group, the Foxp3 mRNA expression level was significantly downregulated in the OVA group and significantly upregulated when anti-IL-17 Abs **G**. were administered. *** *P* < 0.05.

To further evaluate the effects of anti-IL-17 Ab treatment on allergic inflammation, we measured the nasal mucosal levels of mRNAs encoding Th1, Th2 and Th17 cell-related cytokines and Th1, Th2, Th17, and Treg cell-specific transcription factors using real-time PCR (Figure 5). The levels of mRNAs encoding *Ifn-γ,* IL-4 and IL-17 were increased in OVA-induced mice compared to the control mice (*P* < 0.05), and were downregulated upon treatment with anti-IL-17 Abs (*P* < 0.05).

The levels of mRNAs encoding GATA-3 and RORγt were also significantly higher in OVA-induced mice compared to the control mice (*P* < 0.05), and were significantly lower in mice treatment with anti-IL-17 Abs (*P* < 0.05).

The level of mRNAs encoding Foxp3 was significantly decreased in OVA-induced mice compared to the control mice (*P* < 0.05), and was significantly upregulated in mice treatment with anti-IL-17 Abs (*P* < 0.05).

The levels of mRNAs encoding *T-bet* was not significantly changed in OVA-induced mice compared to the control mice (*P* > 0.05), and also was unchanged significantly in mice treatment with anti-IL-17 Abs (*P* > 0.05).

These results suggest that the blockade of IL-17 reduced allergic inflammation by inhibiting the expression of Th2 and Th17 cell-related cytokines and of transcription factors specific to such cells, and that it increased the levels of transcription factors specific to Treg cells.

### Effect of an IL-17 blockade on Th1, Th2, Th17, and Treg cell proportions in nasal mucosa

**Figure 6:**
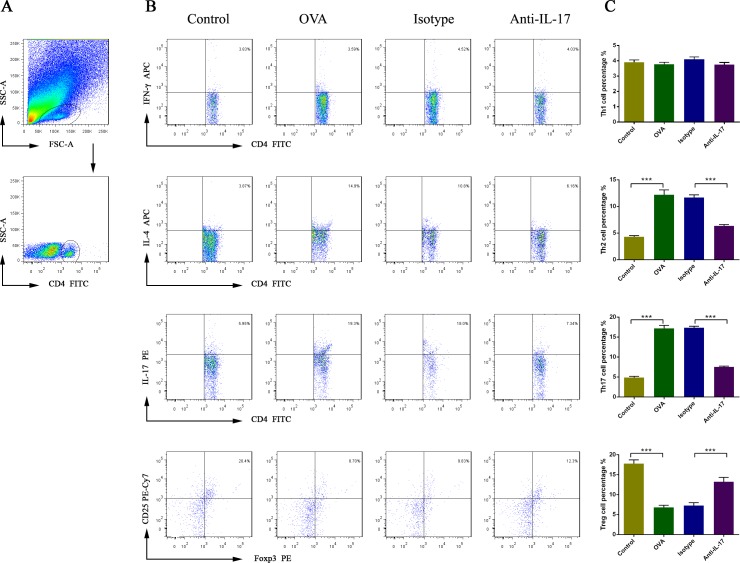
The effects of anti-IL-17 Ab treatment on the proportions of Th1, Th2, Th17, and Treg cells in nasal mucosa, calculated using flow cytometry Gating of CD4^+^ T cell populations **A.** All data are representative of the results of four independent experiments **B**.; the statistics are shown in **C**. The numbers in the upper right quadrants are the proportions of Th1, Th2, Th17, and Treg cells among gated populations of CD4^+^ T cells (B). *** *P* < 0.05.

To evaluate the effects of anti-IL-17 Ab treatment on the lymphocytic immune response, we measured the nasal mucosal proportions of Th1, Th2, Th17, and Treg cells via flow cytometry (Figure 6). The Th2 and Th17 cell proportions were higher in OVA-induced mice than the control mice (*P* < 0.05). After treatment with anti-IL-17 Abs, these proportions fell significantly (*P* < 0.05).

The Th1 cell proportion was not significantly changed in OVA-induced mice compared to the control mice (*P* > 0.05). After treatment with anti-IL-17 Abs, its proportion was unchanged significantly (*P* > 0.05).

The Treg cell proportion was reduced in OVA-induced mice compared to the control mice (*P* < 0.05). After treatment with anti-IL-17 Abs, this proportion increased significantly (*P* < 0.05).

These results indicate that the Th2 and Th17 cell proportions increased, but that of Treg cells decreased, upon AR induction. Following anti-IL-17 treatment, opposite effects were evident. Thus, Th2, Th17, and Treg cells play important roles in the lymphoid immunity associated with AR.

## DISCUSSION

IL-17 expression in the nasal mucosa was associated with nasal eosinophilia and the clinical severity of AR [15]. IL-17 mediates tissue inflammation by inducing the production (by structural cells) of chemokines and proinflammatory cytokines, and it potentiates allergic inflammation by regulating innate immunity [16]. IL-17 can induce allergen-specific Th2 cell activation, eosinophil and neutrophil accumulation, and serum IgE production in asthma [17].

We considered the relationship between IL-17 and eosinophil /neutrophil levels, and the important roles played by eosinophils and neutrophils in AR, and blocked IL-17 action to explore whether eosinophil and neutrophil levels would be affected. We found that IL-17-neutralizing Abs significantly reduced eosinophil and neutrophil levels and significantly improved AR symptoms. These results are consistent with previous study that investigated the immunomodulatory effects of IL-23 and IL-17 in a mouse model of AR [14].

Wang et al.[14]reported that blocked IL-17 cannot reduce significantly sneezes and nasal rubs, however, in our study, we found that increased sneezes and nasal rubs in OVA-induced mice were diminished by treatment with anti-IL-17 Abs, the difference may be because of the different establishment OVA-induce AR model protocol and the different phase for administration anti-IL-17 Abs.

We evaluate the effect of IL-17 blockade on Th1, Th2, Th17 and Treg response. Th1 cell percentage and T-bet mRNA level were both not significantly changed by anti-IL-17 treatment. Th2 responses (the Th2 cell proportion and the levels of IL-4 mRNA/protein, IL-5 protein, IL-13 protein, and GATA-3 mRNA) were increased in OVA-induced mice compared to controls and were downregulated upon anti-IL-17 treatment. These results are also consistent with IL-17 inducing antigen-specific Th2 cell activation [17]. Increased Th17 responses have been recorded in models of asthma, AR, and nasal polyps [7, 17–19]. Consistent with previous reports [3, 17], we found that Th17 responses (the Th17 cell proportion and the levels of IL-17 mRNA/protein and RORγt mRNA) were increased in OVA-induced mice compared to controls, and that they became downregulated upon anti-IL-17 Ab treatment. Unlike other effector CD4+ T cells, Treg cells have been implicated in the peripheral tolerance that inhibits immune responses. Previous studies found that Foxp3 mRNA expression levels were reduced in AR subjects [9–11]. We found that the Treg response (the Treg cell proportion and foxp3 mRNA level) was reduced in OVA-induced mice compared to controls, and became upregulated upon anti-IL-17 Ab treatment.

So, anti-IL-17 Abs can suppress Th2 and Th17 reactions, increase the Treg response. Signal transduction and transcription activating factors (STATs) are a group of intracellular transcription factors, and they play a major role in the signaling pathways Th cell subsets. STAT3 can enhance Th2 cell development in the context of STAT6 signaling [20]. STAT3 also can mediate the development of Th17 cells [21], and the absence of STAT3 increases the proportion of Foxp3 cells expressing T [22]. STAT5, negative regulation of Th17 cell differentiation [21], is also responsible for Treg cell differentiation [23]. We can see that the STAT3/STAT5 ratio can determine the Th17 cells and Treg cells.

While, IL-1β synergizes with IL-23 and IL-6 to regulate Th17 cell differentiation, in which IL-23 and IL-6 can enhance the amplitude and duration of phospho-STAT3 activity, while IL-1β can alter the ratio of STAT3/STAT5 [24], and to maintain cytokine expression in Th17 response [25].

We found that in OVA-induced mice IL-1β, IL-23 and IL-6 were increased which is consistent with previous study [13, 14, 26]. After anti-IL-17 administration, the level of IL-1β, IL-23 and IL-6 decreased(Figure 4F-4H). Through the decreasing of IL-1β, IL-23 and IL-6 levels, so we speculate that anti-IL-17 can inhibit STAT3 activation to inhibit Th2 cell differentiation; change the ratio of STAT3/STAT5 to inhibit Th17 differentiation, meanwhile increase Foxp3 expression so as to improve the Treg content [27, 28], causing corresponding secreted factor changes and alleviate inflammatory reaction.

Although IL-17 is expressed most abundantly by Th17 cells, this cytokine is also produced by other immune cells, including macrophages, B cells, NKT cells, innate lymphoid cells, and CD8^+^ T cells [29–31]. Further work is needed to determine the levels of IL-17 produced by such cells and whether these cells play roles in AR. There was a study showing that the Th17 response enhances not only neutrophilic but also eosinophilic inflammation in mice with AR by using the adoptive transfer of Th17 cells, which may be attributable to local upregulation of eotaxin-1 and -2 expression [29, 32]. However, the mechanisms underlying airway eotaxin induction by Th17 cells remains to be elucidated.

## CONCLUSIONS

We found that the protective effect of anti-IL-17 Ab treatment against AR was mediated by a decrease in eosinophil and neutrophil infiltration, reduced Th2 and Th17 responses, and an increased Treg response. The underlying mechanism may contribute to the modulation of Th2, Th17 and Treg via inhibiting the STAT3 and increasing the STAT5 signaling pathway. These data suggest that the IL-17/Th17/Treg combination plays an important pathogenic role in AR. Our results improve our understanding of AR pathogenesis and may lead to the development of novel therapeutic approaches toward management of the disease.

## MATERIALS AND METHODS

### Animals

Eight-week-old female BALB/c mice, free of murine-specific pathogens, were obtained from the animal department of Shengjing Hospital, China Medical University (Shenyang, China). The mice were housed in a controlled environment under a 12-h/12-h light/dark cycle with free access to food and water. They were maintained on an ovalbumin (OVA)-free diet. All experimental procedures were approved by the ethics committee of Shengjing Hospital, China Medical University.

### Sensitization and antigen challenge

The mice were divided into four treatment groups of ten mice each, as follows: (1) a control group, sensitized and challenged with saline; (2) an OVA group, sensitized and challenged with OVA ; (3) an isotype group treated with isotype Abs for anti-IL-17; and (4) an anti-IL-17 group treated with anti-IL-17 Abs.

The OVA group mice were sensitized on days 0, 2, 4, 6, 8, 10, 12, and 14 via an intraperitoneal injection of 1 mg/mL of OVA (Sigma-Aldrich, St. Louis, MO, USA) and 20 mg/mL of aluminum hydroxide (Sigma-Aldrich) in saline (100 μL/mouse). After 2 weeks, all animals were challenged by the daily nasal instillation of 100 μg of OVA in 20 μL of saline using a micropipette to day 25.

The isotype and anti-IL-17 Ab-treated mice were subjected to the intranasal instillation of mouse IgG (the isotype antibody control for anti-IL-17; eBioscience, San Diego, CA, USA) and anti-IL-17 Abs (eBioscience), respectively, 30 min prior to each OVA challenge; each mouse received 10 μg of antibodies in 20 μL of saline. The control mice were sensitized and challenged with saline instead of OVA.

### Evaluation of allergic symptoms induced after the allergen challenge

On day 25, during the 10-min period after the final administration of intranasal OVA, the numbers of nasal rubbing motions and sneezes were recorded by four observers blinded to the experimental groups. The average values of measurements made by the four observers were used in a statistical analysis.

### Histopathology

Two hours after the last OVA challenge on day 25, all mice were sacrificed. The heads of five mice from each group were removed and immersed in 4% (v/v) buffered paraformaldehyde. After fixation, the heads were decalcified in 10% (w/v) ethylenediaminetetraacetic acid for 28 days. The specimens were embedded in paraffin wax and sectioned coronally at a thickness of 4 μm. The sections were stained with hematoxylin and eosin (H&E) to allow the visualization of eosinophils, which were counted in four randomly selected fields by two observers blinded to the treatments. The mean values of the counts made by the two observers were used in a statistical analysis.

### Collection of nasal mucosa

The nasal mucosae of the remaining five mice from each group were collected and divided into two samples. One sample was placed into RPMI 1640 medium (Invitrogen, Shanghai, China), crushed using a pestle, and filtered through a cell strainer (mesh size 70 um, BD Biosciences, Franklin Lakes, NJ, USA) to obtain single-cell suspensions. After the single-cell suspensions were centrifuged, the supernatants were stored at -80°C prior to cytokine measurement, while the pellets were subjected to analyses of the proportions of neutrophil, Th1, Th2, Th17, and Treg cells. The other sample was immediately snap-frozen in liquid nitrogen and stored at -80°C prior to RNA extraction.

### RNA extraction/reverse transcription and real-time polymerase chain reaction (PCR)

Total RNA was extracted using TRIzol reagent (Invitrogen), and 0.5-μg amounts were reverse-transcribed to complementary DNA using a PrimeScript RT kit (Takara, Dalian, China) according to the manufacturer's protocol. Real-time PCR was performed on an ABI 7500 Real-Time PCR System (Applied Biosystems, Foster City, CA, USA) using SYBR Premix Ex Taq (Takara).

The gene encoding β-actin (a housekeeping gene) served as a control; the genes encoding *Ifn-γ*, IL-4, IL-17A, *T-bet*, gata3, RORγt, and Foxp3 were the target genes. The primer sequences used (written in the 5′-to-3′direction) were as follows: forward primer GCAGAAGGAGATTACTGCTCT, reverse primer GCTGATCCACATCTGCTGGAA for β-actin; forward primer CTGCTGATGGGAGGAGATGT, reverse primer TTTGTCATTCGGGTGTAGTCA for *Ifn-γ*; forward primer TGTACCAGGAGCCATATCCA, reverse primer TGTTCTTCGTTGCTGTGAGG for IL-4; forward primer TCTCTGATGCTGTTGCTGCT, reverse primer CGTGGAACGGTTGAGGTAGT for IL-17A; forward primer TACAACAGCCAGCCAAACAG, reverse primer CACCCTTCAAACCCTTCCTC for *T-bet*; forward primer TACCACCTATCCGCCCTATG, reverse primer GCCTCGACTTACATCCGAAC for gata3; forward primer AGCCTTTCCCTTTCTGCACT, reverse primer CCATCACTTGCTGCTGTTGT for RORγt; and forward primer GCCAAGCAGAAAGATGACAG, reverse primer TTCCAGATGTTGTGGGTGAG for Foxp3.

All mRNA levels were measured using the cycle threshold (2^−ΔΔCT^) method and normalized to those of β-actin. A no-template sample served as a negative control.

### Measurement of cytokine concentrations

The levels of IFN-γ, IL-1β, IL-4, IL-5, IL-6, IL-13, IL-17A and IL-23, in the supernatants were measured using a Cytometric Bead Array (CBA) Flex Set (BD Biosciences) according to the manufacturer's instructions. Briefly, capture bead populations differing in terms of fluorescence intensities, coated with cytokine-specific capture antibodies, were mixed together in equal volumes. A 50-μL aliquot of each sample and 50 μL of a mixture of phycoerythrin (PE)-conjugated detection antibodies were added to 50 μL of the mixed-bead population. Each mixture was incubated for 3 h at room temperature in the dark to allow sandwich complexes to form. Next, the beads were washed with wash buffer and data were acquired using a BD FACS Canto II flow cytometer (BD Biosciences) running FACSDiva and BD CBA software ver. 4.2 (BD Biosciences).

The limits of detection were 0.5 pg/mL for IFN-γ, 2 pg/mL for IL-1β, 0.3 pg/mL for IL-4, 0.9 pg/mL for IL-5, 3 pg/mL for IL-6, 2.4 pg/mL for IL-13, 0.95 pg/mL for IL-17A and 5 pg/mL for IL-23. Zero values were assigned when the levels were under these limits.

### Flow cytometric analysis of the neutrophil proportion

Cells were stained with fluorescein isothiocyanate (FITC)-labeled anti-Gr-1 Abs and PE-labeled anti-CD11b Abs (BD Biosciences). All samples were analyzed on a BD FACSCanto II flow cytometer (BD Biosciences); the data were evaluated using FlowJo software (ver. 7.6; TreeStar Inc.).

### Flow cytometric analysis of the Th1, Th2 and Th17 cell proportions

Cells were stimulated with 50 ng/mL of phorbol myristate acetate (Sigma-Aldrich), 1 μg/mL of ionomycin (Sigma-Aldrich), and 10 μg/mL of GolgiStop (BD Biosciences) at 37°C under 5% (v/v) CO2 for 6 h, and then stained with fluorescein isothiocyanate-labeled anti-CD4 Abs (BD Biosciences). The cells were next fixed and permeabilized using a fix/perm solution (eBioscience) according to the manufacturer's instructions. The cells were next incubated with allophycocyanin (APC)-labeled IFN-γ Abs, APC-labeled IL-4 Abs and PE-labeled IL-17A Abs (BD Biosciences). Cells in the control group were stained with isotype control Abs. The samples were analyzed on a BD FACSCanto II flow cytometer (BD Biosciences); the data were evaluated using FlowJo software (ver. 7.6; TreeStar Inc., San Carlos, CA, USA).

### Flow cytometric analysis of the Treg cell proportions

Cells were stained with fluorescein isothiocyanate (FITC)-labeled anti-CD4 Abs and PE-cyanin (Cy)7-labeled anti-CD25 Abs (BD Biosciences), and then fixed and permeabilized using a fix/perm solution (eBioscience) according to the manufacturer's instructions. The cells were then incubated with PE-labeled anti-Foxp3 Abs (BD Biosciences); control cells were incubated with the Foxp3 isotype control. All samples were analyzed on a BD FACSCanto II flow cytometer (BD Biosciences); the data were evaluated using FlowJo software (ver. 7.6; TreeStar Inc.).

### Statistical analysis

All results are expressed as means ± SEMs. Among-group comparisons were made using the non-parametric Kruskal-Wallis test, followed by the Mann-Whitney U test. All statistical analyses were performed with the aid of SPSS software (ver. 13.0; SPSS Inc., Chicago, IL, USA). *P*-values < 0.05 were considered to indicate statistical significance.

## References

[1] Skoner DP Allergic rhinitis: definition, epidemiology, pathophysiology, detection, and diagnosis. J Allergy Clin Immunol.

[2] Malmhäll C, Bossios A, Pullerits T, Lötvall J Effects of pollen and nasal glucocorticoid on FOXP3+, GATA-3+ and T-bet+ cells in allergic rhinitis. Allergy.

[3] Wang SB, Deng YQ, Ren J, Xiao BK, Liu Z, Tao ZZ Exogenous interleukin-10 alleviates allergic inflammation but inhibits local interleukin-10 expression in a mouse allergic rhinitis model. BMC Immunol.

[4] Infante-Duarte C, Horton HF, Byrne MC, Kamradt T Microbial lipopeptides induce the production of IL-17 in Th cells. J Immunol.

[5] Ivanov II, McKenzie BS, Zhou L, Tadokoro CE, Lepelley A, Lafaille JJ, Cua DJ, Littman DR The orphan nuclear receptor RORgammat directs the differentiation program of proinflammatory IL-17+ T helper cells. Cell.

[6] Wang SB, Deng YQ, Ren J, Xiao BK, Chen Z, Tao ZZ Lactoferrin administration into the nostril alleviates murine allergic rhinitis and its mechanisms. Scand J Immunol.

[7] Wakashin H, Hirose K, Maezawa Y, Kagami S, Suto A, Watanabe N, Saito Y, Hatano M, Tokuhisa T, Iwakura Y, Puccetti P, Iwamoto I, Nakajima H IL-23 and Th17 cells enhance Th2-cell-mediated eosinophilic airway inflammation in mice. Am J Respir Crit Care Med.

[8] Wilson RH, Whitehead GS, Nakano H, Free ME, Kolls JK, Cook DN Allergic sensitization through the airway primes Th17-dependent neutrophilia and airway hyperresponsiveness. Am J Respir Crit Care Med.

[9] Mo JH, Chung YJ, Kim JH T cell transcriptional factors in allergic rhinitis and its association with clinical features. Asia Pac Allergy.

[10] Xu G, Mou Z, Jiang H, Cheng L, Shi J, Xu R, Oh Y, Li H A possible role of CD4+CD25+ T cells as well as transcription factor Foxp3 in the dysregulation of allergic rhinitis. Laryngoscope.

[11] Lee SM, Gao B, Dahl M, Calhoun K, Fang D Decreased FoxP3 gene expression in the nasal secretions from patients with allergic rhinitis. Otolaryngol Head Neck Surg.

[12] Ciprandi G, De Amici M, Murdaca G, Fenoglio D, Ricciardolo F, Marseglia G, Tosca M Serum interleukin-17 levels are related to clinical severity in allergic rhinitis. Allergy.

[13] Moon IJ, Hong SL, Kim DY, Lee CH, Rhee CS, Min YG (2012). Blocking interleukin-17 attenuates enhanced inflammation by staphylococcal enterotoxin B in murine allergic rhinitis model. Acta Otolaryngol.

[14] Wang M, Zhang W, Shang J, Yang J, Zhang L, Bachert C Immunomodulatory effects of IL-23 and IL-17 in a mouse model of allergic rhinitis. Clin Exp Allergy.

[15] Makihara S, Okano M, Fujiwara T, Noda Y, Higaki T, Miyateke T, Kanai K, Haruna T, Kariya S, Nishizaki K Local expression of interleukin-17a is correlated with nasal eosinophilia and clinical severity in allergic rhinitis. Allergy Rhinol (Providence).

[16] Wang YH, Liu YJ The IL-17 cytokine family and their role in allergic inflammation. Curr Opin Immunol.

[17] Quan SH, Zhang YL, Han DH, Iwakura Y, Rhee CS Contribution of interleukin 17A to the development and regulation of allergic inflammation in a murine allergic rhinitis model. Ann Allergy Asthma Immunol.

[18] Zhao J, Lloyd CM, Noble A Th17 responses in chronic allergic airway inflammation abrogate regulatory T-cell-mediated tolerance and contribute to airway remodeling. Mucosal Immunol.

[19] Cao PP, Li HB, Wang BF, Wang SB, You XJ, Cui YH, Wang DY, Desrosiers M, Liu Z Distinct immunopathologic characteristics of various types of chronic rhinosinusitis in adult Chinese. J Allergy Clin Immunol.

[20] Stritesky GL, Muthukrishnan R, Sehra S, Goswami R, Pham D, Travers J, Nguyen ET, Levy DE, Kaplan MH The transcription factor STAT3 is required for T helper 2 cell development. Immunity.

[21] Laurence A, Tato CM, Davidson TS, Kanno Y, Chen Z, Yao Z, Blank RB, Meylan F, Siegel R, Hennighausen L, Shevach EM, O'shea JJ Interleukin-2 signaling via STAT5 constrains T helper 17 cell generation. Immunity.

[22] Yao Z, Kanno Y, Kerenyi M, Stephens G, Durant L, Watford WT, Laurence A, Robinson GW, Shevach EM, Moriggl R, Hennighausen L, Wu C, O'Shea JJ Nonredundant roles for Stat5a/b in directly regulating Foxp3. Blood.

[23] Lochner M, Peduto L, Cherrier M, Sawa S, Langa F, Varona R, Riethmacher D, Si-Tahar M, Di Santo JP, In Eberl G vivo equilibrium of proinflammatory IL-17+ and regulatory IL-10+ Foxp3+ RORgamma t+ T cells. J Exp Med.

[24] Basu R, Whitley SK, Bhaumik S, Zindl CL, Schoeb TR, Benveniste EN, Pear WS, Hatton RD, Weaver CT IL-1 signaling modulates activation of STAT transcription factors to antagonize retinoic acid signaling and control the TH17 cell-iTreg cell balance. Nat Immunol.

[25] Chung Y, Chang SH, Martinez GJ, Yang XO, Nurieva R, Kang HS, Ma L, Watowich SS, Jetten AM, Tian Q, Dong C Critical regulation of early Th17 cell differentiation by interleukin-1 signaling. Immunity.

[26] Guo C, Chen G, Ge R IL-23, rather than IL-17, is crucial for the development of ovalbumin-induced allergic rhinitis. Mol Immunol.

[27] Ju JH, Heo YJ, Cho ML, Jhun JY, Park JS, Lee SY, Oh HJ, Moon SJ, Kwok SK, Park KS, Park SH, Kim HY (2012). Modulation of STAT-3 in rheumatoid synovial T cells suppresses Th17 differentiation and increases the proportion of Treg cells. Arthritis Rheum.

[28] Jhun J, Lee J, Byun JK, Kim EK, Woo JW, Lee JH, Kwok SK, Ju JH, Park KS, Kim HY, Park SH, Cho ML (2014). Red ginseng extract ameliorates autoimmune arthritis via regulation of STAT3 pathway, Th17/Treg balance, and osteoclastogenesis in mice and human. Mediators Inflamm.

[29] Liu Y, Zeng M, Liu Z Th17 response and its regulation in inflammatory upper airway diseases. Clin Exp Allergy.

[30] Schmidt-Weber CB, Akdis M, Akdis CA TH17 cells in the big picture of immunology. J Allergy Clin Immunol.

[31] Miossec P, Korn T, Kuchroo VK Interleukin-17 and type 17 helper T cells. N Engl J Med.

[32] Liu Y, Yu HJ, Wang N, Zhang YN, Huang SK, Cui YH, Liu Z Clara cell 10-kDa protein inhibits T(H)17 responses through modulating dendritic cells in the setting of allergic rhinitis. J Allergy Clin Immunol.

